# Molecular Phylogenomics Reveals the Deep Evolutionary History of Carnivory across Land Plants

**DOI:** 10.3390/plants12193356

**Published:** 2023-09-22

**Authors:** Steven J. Fleck, Richard W. Jobson

**Affiliations:** 1 Department of Biological Sciences, University at Buffalo, Buffalo, NY 14260, USA; 2 National Herbarium of New South Wales, Botanic Gardens of Sydney, Locked Bag 6002, Mount Annan, NSW 2567, Australia

**Keywords:** Phylogenomics, Caryophyllales, Ericales, genomics, Lamiales, Oxidales, Poales, Alismatales

## Abstract

Plastid molecular phylogenies that broadly sampled angiosperm lineages imply that carnivorous plants evolved at least 11 times independently in 13 families and 6 orders. Within and between these clades, the different prey capture strategies involving flypaper and pitfall structures arose in parallel with the subsequent evolution of snap traps and suction bladders. Attempts to discern the deep ontological history of carnivorous structures using multigene phylogenies have provided a plastid-level picture of sister relationships at the family level. Here, we present a molecular phylogeny of the angiosperms based on nuclear target sequence capture data (Angiosperms-353 probe set), assembled by the Kew Plant Trees of Life initiative, which aims to complete the tree of life for plants. This phylogeny encompasses all carnivorous and protocarnivorous families, although certain genera such as *Philcoxia* (Plantaginaceae) are excluded. This study offers a novel nuclear gene-based overview of relationships within and between carnivorous families and genera. Consistent with previous broadly sampled studies, we found that most carnivorous families are not affiliated with any single family. Instead, they emerge as sister groups to large clades comprising multiple non-carnivorous families. Additionally, we explore recent genomic studies across various carnivorous clades that examine the evolution of the carnivorous syndrome in relation to whole-genome duplication, subgenome dominance, small-scale gene duplication, and convergent evolution. Furthermore, we discuss insights into genome size evolution through the lens of carnivorous plant genomes.

## 1. Introduction

The first Sanger-sequenced molecular phylogeny using the plastid *rbcL* gene across all major angiosperm lineages provided an opportunity to map the evolution of carnivory, and uncovered evidence of at least five independent origins of carnivorous plants [1]. Since that study, additional cases of carnivory have been verified for members of the Poales families Bromeliaceae (*Brocchinia hechtiodes*, *B. reducta* [2,3], *Catopsis beteronianum* [4]) and Eriocaulaceae (*Paepalanthus bromelioides* [5]), in the Ericales (*Roridula gorgonias*) [6], and Lamiales (Plantaginaceae; *Philcoxia* spp. [7,8]) (reviewed in [9,10]), and most recently in the Alismatales family Toldfeldiaceae (*Triantha occidentalis*) [11,12]. A newly discovered and described African species, *Crepidorhopalon droseroides* (Linderniaceae), is suspected of being carnivorous via a flypaper strategy on the leaves, with future study required to determine whether nutrient uptake occurs [13]. Across 6 angiosperm orders, there are 829 recognized carnivorous species in 21 genera and 13 families [8,9,12]. Currently, the evolution of carnivory is known to have arisen independently at least 12 times: four times in the monocots (Alismatales and Poales), once in the Rosids (Oxidales), and seven times in the superasterides (Caryophyllales, Ericales, Lamiales) ([8,9,12], for order and family descriptions refer to [14]).

**Figure 1:**
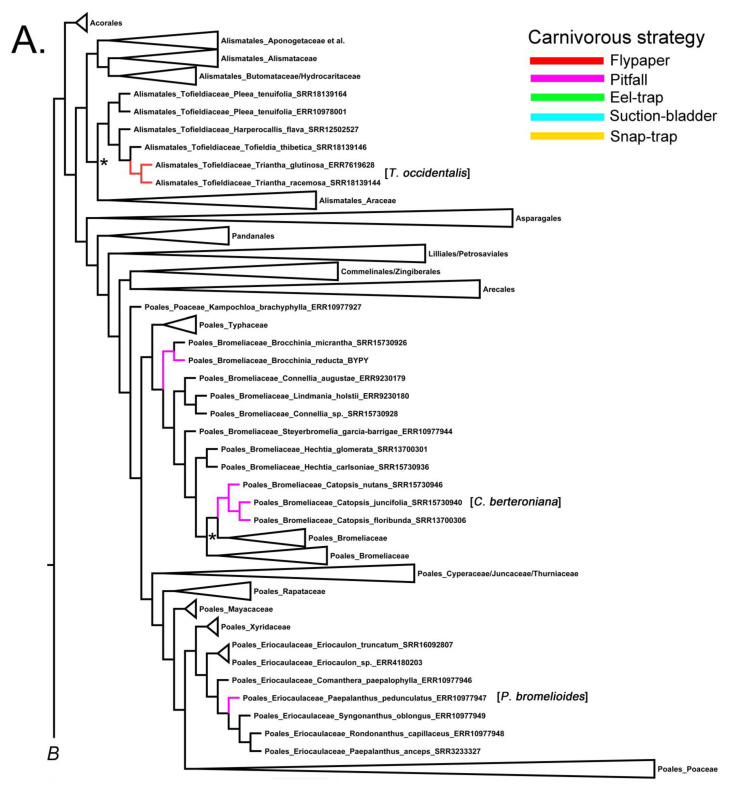

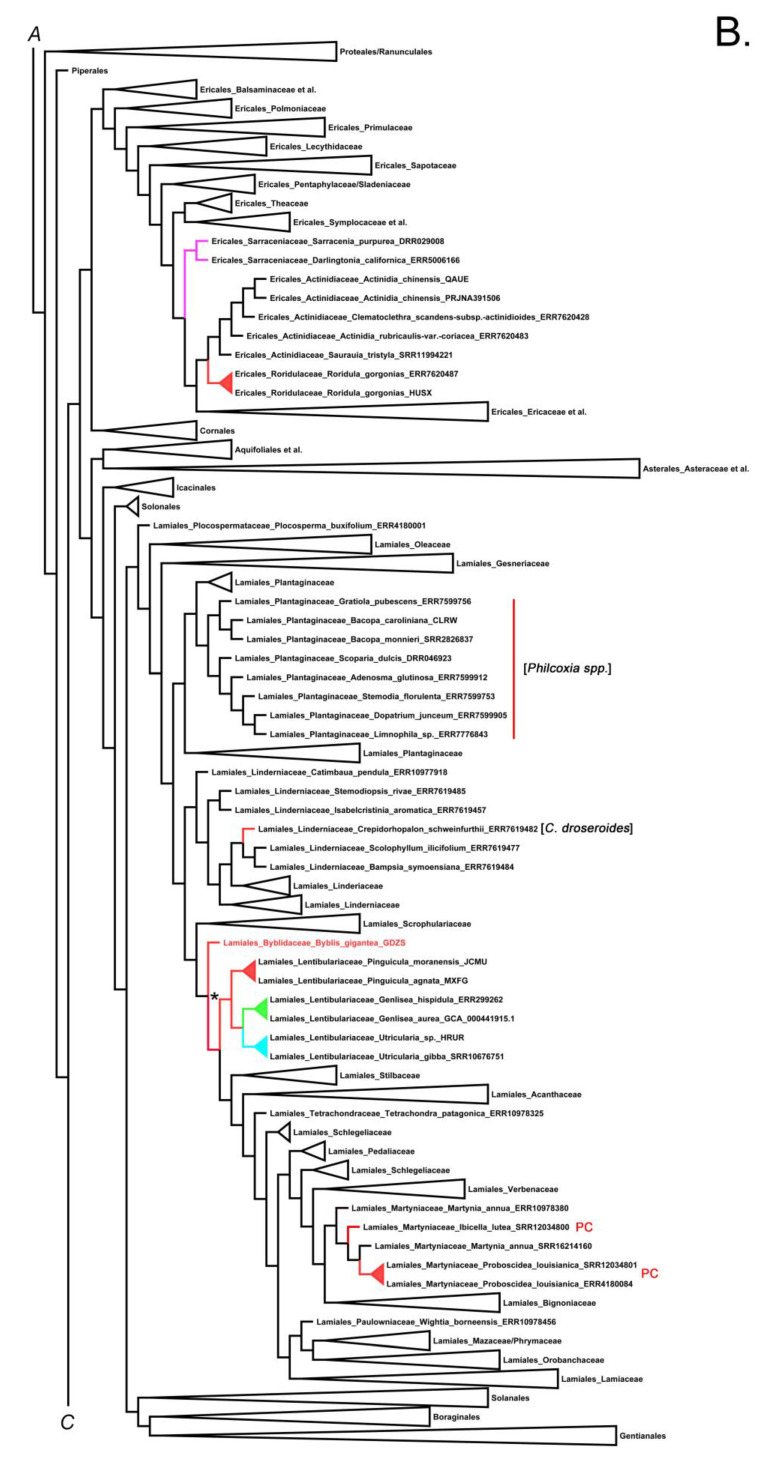

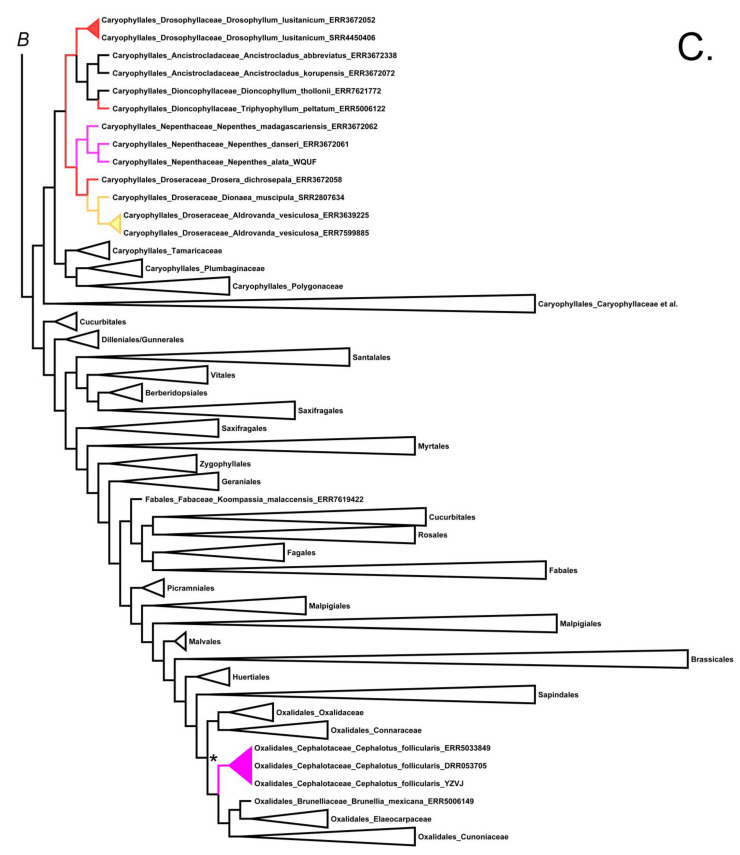
Fifty percent majority-rule Bayesian inference consensus tree reconstructed from Aangiosperms-353 target capture data downloaded from the Kew Tree of Life Explorer Version 3.0 released in April 2023 (https://treeoflife.kew.org/) and trees (accessed on 20 July 2023). (**A**) Monocot clades; (**B**), Ericales and Lamiales clades; and (**C**) Caryophyllales and Oxidales clades. Branches and clades were collapsed or modified using FigTree (ver. 1.4.0, see http://tree.bio.ed.ac.uk/software/figtree/, accessed 20 July 2023). Branches are color-coded according to the carnivorous strategy (see legend). Approximate position of missing carnivorous taxa shown in brackets. Relevant non-supported nodes (posterior probability < 0.96) shown with asterisks. Strong node support 0.96–1.0 not shown. PS = protocarnivorous taxon.

The flypaper and pitfall strategies are perhaps the least developmentally complex, with each evolving seven times across angiosperms (Figure 1A–C). The flypaper or adhesive strategy involves the modification of glandular structures for the production of sticky mucilage and enzymes [15,16]. The pitfall strategy can involve the utilization of extant structures with the modification of leaf surface glands in water-filled tanks in the Bromeliaceae and Eriocaulaceae (reviewed in [9]), or modified leaf structures with epiascidiate development forming pitcher-like tanks [15,16]. The active suction-trap and passive eel-trap strategies [17] evolved from a common ancestor that utilized the flypaper strategy [18,19], with modified trapping leaves of both genera sharing initial developments pathways [15,17]. The snap trapping strategy has evolved once and diverged into two monotypic genera, with the traps of one operating when submerged in water and the other being terrestrial [17,20].

Within the Lamiales, a fully carnivorous flypaper strategy independently arose in Byblidaceae and Lentibulariaceae, with two additional cases of protocarnivory in the Marytiniaceae ([2], reviewed in [15]). Likewise, in Caryophyllales, the flypaper strategy evolved three times in Drosophyllaceae, Dionocophyllaceae, and Droseraceae (*Drosera*). The family Nepenthaceae is sister to the Droseraceae. Nepenthaceae has modified leaves that form pitcher trap structures. In Droseraceae, the *Drosera* sister taxa are the aquatic *Aldrovanda* and terrestrial *Dionaea*. These taxa have evolved modified leaves with active steel-trap function [17,21]. Attempts to discern the deep ontological history of carnivorous structures using multigene phylogenies has so far mostly failed to determine sister relationships at the family level [9,21]. More recent phylogenetic studies have focused on determining family-level relationships using multigene phylogenies sampled broadly within each of the recognized genera (reviewed in [22]).

In this study, we make use of a molecular phylogeny of the angiosperms derived from nuclear target sequence capture data (Angiosperms-353 probe set). These data were assembled by the Kew Plant Trees of Life initiative (https://www.kew.org/science/our-science/projects/plant-and-fungal-trees-of-life (accessed on 20 July 2023)), which aims to complete the tree of life for plants [23,24,25]. Upon this phylogenetic hypothesis, we map all carnivorous families to explore generic and family relationships among and between carnivorous taxa. We compare these relationships with those of previous plastid phylogenetic studies. We also examine recent genomic studies within various carnivorous clades, exploring the evolution of carnivory and the questions these groups adeptly address.

## 2. Monocots: Poales

### 2.1. Bromeliaceae

Carnivory emerged independently at least twice within the Poales family Bromeliaceae [9]. Two of the 20 species of *Brocchinia* and one of the 19 species of *Catopsis* are considered carnivorous [9,26,27]. All species of *Brocchinia* (monogeneric subfamily Broccinioideae) are endemic to the Guyana Shield, primarily located in wet savannah habitats. In contrast, the neotropical *Catopsis* species (subfamily Tillandsioideae) are epiphytes [26,27]. All three carnivorous species utilize water-filled rosette tanks as pitfall traps where specialized trichomes absorb nutrients from insect prey and debris [2,15].

Previous phylogenetic studies found Bromeliaceae sister to Typhaceae, and together they are sister to all other Poales families [28,29,30], agreeing with the current phylogeny (Figure 1A). In Figure 1A, a monophyletic *Brocchinia* is placed at the base of the family, sister to all sampled Bromeliaceae genera [28]. Of the two carnivorous species, Figure 1A includes *B. reducta*, along with the non-carnivorous *B. micrantha*. The placement of the other carnivorous species, *B. hechtioides*, which is not included in the current study, meant that it was found to be sister to *B. reducta* ([4], reviewed in [9,27]).

Even though the carnivorous *Catopsis berteroniana* is not included in the current study, a previous phylogenetic study showed that it nests within a monophyly of the genus [26]. Figure 1A illustrates that three non-carnivorous species exhibit the same intergeneric relationship between *Brochinnia* and *Catopsis*, as observed in previous studies based on plastid DNA sequences. This further supports the independent evolution of carnivory in Bromeliacae [2,3,4,27,28].

### 2.2. Eriocaulaceae

*Paepalanthus* contains between 300–400 spp. mostly distributed in tropical Africa and the neotropics [31,32]. The Brazilian *P. bromelioides* forms a water-holding tank within its rosette of leaves and is currently considered a carnivore based on δ 15 N values that indicate nitrogen uptake from animal prey. Experimental demonstrations of nutrient absorption are yet to be executed [27]. The upper leaf surface is waxy with glandular structures present towards the base [5]. The fully supported Eriocaulaceae is sister to the fully supported Xyridaceae, and together they are sister to the Restionaceae + Poaceae and allied families (Figure 1A). Although *P. bromelioides* is not represented in the current tree, it was included in a phylogeny showing monophyly of the genus [31] within the same clade as our represented taxon *P. pedunculatus* (Figure 1A). 

## 3. Monocots: Alismatales

### Toldfeldiaceae

The present phylogeny reveals that Alismatales comprise two well-supported major clades: one primarily composed of aquatic lineages and the other consisting of the monophyletic sister clades Toldfeldiaceae and Araceae (Figure 1A). However, posterior support for this relationship is equivocal (Figure 1A), and [11]—using plastome sequences and a much denser sampling of genera in Alismatales—inferred that Tofieldiaceae is more likely sister to both Araceae and the aquatic lineage of Alismatales. Within the Toldfeldiaceae, the sister genera *Toldfeldia* (12 spp.) and *Triantha* (4 spp.) are fully supported (Figure 1A). Thus far, only *Triantha occidentalis* has been demonstrated to uptake N from prey capture via sticky glands on its inflorescence [12]. While *T. occidentalis* is not included in the current sampling of the genus, we highlight the two represented species as proxies (Figure 1A).

## 4. Superasterids: Lamiales

### 4.1. Plantaginaceae and Linderniaceae

The Brazilian endemic genus, *Philcoxia*, contains seven species [7,8,27,33], all possessing the flypaper strategy. It has sticky glands on small peltate leaves that are held at the surface or just below the surface of a sandy substrate that presumably function in the capture of nematodes [34]. Although initial tests for protease activity on leaf surfaces were negative, the obvious specialization for prey capture led Fritsch et al. [35] to suggest the strong possibility of carnivory. This was confirmed by Pereira et al. [34] using isotopic analysis that found that nitrogen derived from nematode prey was assimilated into plant tissue.

*Philcoxia* was initially thought to have an affinity with the tribe Gratioleae (Plantaginaceae), and recent phylogenetic analyses support this placement as sister to the genus *Lapaea* [36,37]. Although an accession of *Philcoxia* was not included in the 353 dataset, the branching order shows the Plantaginaceae to be sister to the Linderniaceae and both as being sister to the Scropulariaceae (Figure 1B). A future Angiosperm-353 study, including the addition of *Philcoxia* and a more comprehensive sampling of Gratioleae genera, particularly *Lapaea*, is required. 

The phylogenetic position of the potentially new flypaper-trapping taxon *Crepidorhopalon droseroides* (Linderniaceae) [13] is shown in Figure 1B using a related species as a generic proxy. If carnivory is confirmed for this taxon, its evolution would represent an additional independent evolution of the flypaper-trapping strategy. 

### 4.2. Byblidaceae and Lentibulariaceae

In Figure 1B, a monophyletic clade of Schrophulariaceae is followed by a single accession representing the Australasian genus *Byblis* that contains eight recognised species (Byblidaceae). Byblidaceae is sister to a large clade of families, with carnivorous Lentibulariaceae being sister to all others in that clade. This family includes three genera (Figure 1B), each employing distinct prey capture strategies [15]. Figure 1B illustrates that there is no branch support at the node between Byblidaceae and Lentibulariaceae. This suggests the possibility of both families falling into the same clade, which is consistent with findings from the three and two chloroplast marker phylogenies of Bremer et al. [38] and Jobson et al. [18], respectively. Conversely, a single-marker study of Lentibulariaceae conducted by Muller et al. [39] employed a broader sampling across the Lamiales. This study positioned Byblidaceae in proximity to Linderniaceae and Stilbaceae, while Lentibulariaceae was situated in a separate, distant clade, which was sister to Bignoniaceae. The result of Muller et al. [39] corresponds closely with that of Li et al. [40], who used full chloroplast genomes and found *Byblis* to be sister to Linderniaceae, while Lentibulariaceae was sister to Thomandersiaceae in a clade with Martyniaceae. The reason for the discordance between chloroplast genomes and the current nuclear tree requires further investigation.

The current tree places Lentibulariaceae closest to Stilbaceae, succeeded by the sister clades Acanthaceae, Schlegeliaceae, and Pedaliaceae. These are followed by Verbenaceae, Martyniaceae + Bignoniaceae, and Lamiaceae, that are sister to Phrymaceae + Orobanchaceae (Figure 1B). Stilbaceae consists of 12 small genera native to Africa and Madagascar, with few synapomorphies shared between it and Lentibulariaceae [41]. All three genera of the Lentibulariaceae were broadly sampled in phylogenetic studies focused on the boreotropical *Pinguicula* (reviewed in [42]), Paleotropical and Neotropical *Genlisea* (reviewed in [43]), and cosmopolitan *Utricularia* (reviewed in [44]). All previous studies based on plastid DNA sequences showed the same generic branching order as that of the current study with a monophyletic *Pinguicula* sister to a clade of monophyletic genera *Genlisea* and *Utricularia* (Figure 1B). 

In the Lentibulariaceae, carnivory involves flypaper trapping and nutrient-absorbing glands on leaves in *Pinguicula*. This evolution then diverges into two additional carnivorous strategies, both of which share a common developmental ontogeny of modified leaf structures, initially forming epiacidiate invaginations [15]. In *Genlisea*, the invagination develops into bifurcating tubular structures that passively capture subterranean soil organisms. Conversely, in its sister genus *Utricularia*, the tubes are replaced by a hollow bladder structure with a terminal hinged door, facilitating the active pumping of internal fluid. This results in an active suction mechanism used for capturing subterranean and water column organisms [15,17]. For *Utricularia*, several studies suggest that one of the subgeneric lineages, *Polypompholyx*, has inactive bladder traps (reviewed in [9]). However, other studies have presented evidence demonstrating normal bladder activity within this subgenus (reviewed in [44]).

## 5. Superasterids: Ericales

### Sarraceniaceae and Roridulaceae

Sarraceniaceae consists of the monotypic North American *Darlingtonia*, 23 species of neotropical *Heliamphora*, and 11 species of North American *Sarracenia* [45]. All three Sarraceniaceae genera possess the pitfall trapping strategy with tubular structures forming epiascidiate-type fusion of the leaf margin [15,16]. The two South African species of *Roridula* have leaves covered in sticky glandular hairs for trapping prey, although nutrients are utilized via a mutualistic relationship with two species of the Hemipteran genus *Pameridea* [27].

The highly modified floral characteristics of Sarraceniaceae made placement difficult. Early researchers suggested affinity with Ranunculaceae and Papaveraceae [46,47] or Caryophyllaceae [48]. Other researchers suggested an affiliation within Cornales or Ericales [49,50].

Albert et al. [1] provided the first molecular phylogenetic insight into the origins of carnivorous plants using the *rbc*L plastid marker with a broad sampling across angiosperm families. The *Darlingtonia*, *Heliamphora* and *Sarracenia* genera formed a monophyletic Sarraceniaceae, sister to the proto-carnivorous *Rordidula* (Roridulaceae), and together this clade was sister to two accessions from the Ericaceae (Ericales). These relationships were later supported by the inclusion of the nuclear ITS marker by Bayer et al. [51]. Albach et al. [52] used three additional molecular markers and expanded the sampling to include Actinidiaceae, finding it to be sister to Roridulaceae, and Li et al. [40] found the same result using full chloroplast genomes. The current tree supports the above studies, showing strong support for the grouping of Theaceae, Symplocaceae, and Styracaceae + Diapensiaceae as sister to Sarraceniaceae, Roridulaceae + Actinidiaceae, and Ericaceae/Clethraceae/Cyrillaceae (Figure 1B). The phylogenetic relationships within Sarraceniaceae have been studied using mitochondrial, chloroplast, and nuclear DNA markers, placing *Heliamphora* as sister to *Sarracenia*, and together these are sister to the monotypic *Darlingtonia* [53,54]. 

In the present phylogeny (Figure 1B), Sarraceniaceae is represented by a single sample from both *Sarracenia* and *Darlingtonia*. Their placement at the base of the clade, sister to Roridulaceae + Actinidiaceae/Ericaceae, suggests an independent evolution of carnivory within Ericales. This proposition aligns with earlier findings by [54] as outlined in [45]. 

## 6. Superasterids: Caryophyllales

Within the Caryophyllales, carnivory may have evolved once, only to be subsequently lost once or multiple times during the divergence that led to the flypaper-trapping monotypic family Drosophyllaceae, as well as Ancistrocladaceae + Dioncophylaceae [9] (Figure 1C). A subsequent partial gain of flypaper trapping (only exhibited in part of the life cycle) is evident in the monotypic genus *Triphyophyllum* (Dioncophylaceae) [15,27]. The sister clade to all of the above families consists entirely of carnivorous genera in two monophyletic sister clades, Nepenthaceae and Droseraceae (Figure 1C). This relationship was previously reported in a phylogenetic study that sampled broadly across related families [14,40]. Together, Nepenthaceae + Droseraceae are sister to all other Caryophyllales clades, supporting a previous report suggesting early divergence within the order [9]. The next branching clade includes Frankeniaceae + Tamaricaceae followed by monophyletic sister clades Plumbaginaceae + Polygonaceae (Figure 1C). The presence of plumbagin serves as a synapomorphy for this entire group. Additionally, a transition from pollen in monads to tetrads occurred in the ancestor of Nepenthaceae + Droseraceae [9].

The Droseraceae clade contains a single accession representing *Drosera*, a genus containing c. 240 spp. with leaves modified for flypaper trapping, distributed across the world [21]. The sister relationship of monotypic genera *Dionaea* and *Aldrovanda* agrees with the multi-gene phylogeny of Cameron et al. [20]. Both genera share the snap-trapping strategy where modified leaves are stimulated to close upon prey organisms [15,17,55]. The full chloroplast phylogeny of Li et al. [40] found *Drosera* paraphyletic with *D. regia* to be sister to *Dionaea* + *Aldrovanda*, and these together were sister to a clade of *Drosera* species. Cameron et al. [20] also included *D. regia* and found that *matK* and *rbcL* trees showed a paraphyletic *Drosera*, while *atpB* and nuclear 18S each showed a monophyletic *Drosera*. When these four markers were analyzed together, *Drosera* and *Dionaea* + *Aldrovanda* were each supported as being monophyletic [20].

The genus *Nepenthes* employs a passive pitfall-trapping strategy and has c. 150 species distributed across the paleotropics [56,57,58]. Previous phylogenetic studies that sampled broadly across related families [14,40] reinforced the above relationships (Figure 1C).

## 7. Superrosids: Oxalidales

### Cephalotaceae

Carnivory evolved once in the Oxidales, specifically within the monotypic Cephalotaceae family, which is endemic to a few coastal sites in southwestern Western Australia [27]. The earliest molecular phylogeny to include *Cephalotus* placed it as sister to *Oxalis* [1]. Subsequent studies found that it is nested within Oxidales, with some placing it as sister to Brunelliaceae [29,40,59], while others found a sister relationship with Elaeocarpaceae [60]. The current tree shows that the fully supported Oxidales clade is weakly supported as being sister to Huerteales. Oxidales consists of two fully supported major groupings of monophyletic families with Oxalidaceae + Connaraceae sister to Cephalotaceae, Elaeocarpaceae, Cunoniaceae + Brunelliaceae (Figure 1C). Cephalotaceae is the earliest branching member of the above clade, and although these two groupings share no salient vegetative synapomorphies, floral homology is evident [9]. The grouping exhibits considerable variation in terms of biogeography, growth form, and ecology. This variation is evident between Cephalotaceae and the monotypic *Brunellia*, a small genus of 52 neotropical tree species. Elaeocarpaceae consists of 11 genera and 22 species of trees and shrubs from across the tropics. Cunoniaceae encompasses 24 genera and 340 species of trees and shrubs distributed throughout the southern hemisphere [41]. In contrast, the small rosettes of the herbaceous *Cephalotus* can develop into two morphological forms: a typical lanceolate leaf with an entire margin, or a sophisticated epiascidiate pitcher structure, the development of which is still not fully understood [9].

## 8. Carnivorous Plant Genomes

Carnivorous plants provide interesting examples of genome evolution. The species that have been studied so far provide insights into the evolution of carnivory, genome size, polyploidy, gene duplication, and more. The first chromosome-level genome assembly for carnivorous taxa was *Nepenthes gracilis*, the Asian pitcher plant [61]. The only other published carnivorous genome assembly to contain some whole-chromosome pseudomolecules is *Utricularia gibba*, the humped bladderwort [62]. While *N. gracilis* and *U. gibba* are the only (at least partially) chromosome-level genome assemblies to date, other less contiguous and less complete assemblies have been published and have provided important insights into the evolution of carnivory, such as *Genlisea aurea* [63], *G. nigrocaulis*, *G. hispidula* [64], and *Utricularia reniformis* [65] in the Lentibulariaceae (Lamiales), *Roridula gorgonias* [66] in the Roridulaceae (Ericales), *Nepenthes mirabilis* [67] in the Nepenthaceae (Caryophyllales), *Aldrovanda vesiculosa*, *Dionaea muscipula*, *Drosera spatulata* [68] and *Drosera capensis* [69] in the Droseraceae (Caryophyllales), and *Cephalotus follicularis* [70] in the Cephalotaceae (Oxalidales). Here, we discuss some of the recent research on genome evolution in carnivorous plants.

Carnivorous taxa are among the smallest known plant genomes, with members of *Utricularia* and *Genlisea* having haploid genome sizes estimated at under 100 million base pairs (Mbp) [64,71,72,73,74]. With one of the smallest and largest genomes within the genus, respectively, *Genlisea nigrocaulis* (73–86 Mbp) and *G. hispidula* (1417–1550 Mbp) show at least a 16-fold difference in genome size [64,71,72,73,74]. An extra whole-genome duplication (WGD) in *G. hispidula* since it diverged from *G. nigrocaulis* [64] might contribute to some of this difference. Interestingly, *G. aurea* also experienced a WGD after diverging from *G. hispidula* [64], yet its genome is 24 times smaller than *G. hispidula*, measuring only 64–131 Mbp [71,72,73,74]. The discrepancy in size between these genomes is due to the proliferation of transposable elements (TEs) that copy themselves throughout the genome, in the case of *G. hispida*, and the silencing of TEs and a bias towards deletion during DNA double-strand break (DSB) repair, in the case of *G. nigrocaulis* [64] and even more so in *G. aurea* [63]. In addition to a reduction in intergenic regions (including TEs), introns were also reduced in size in both *G. aurea* and *G. nigrocaulis* without reducing the number of introns per gene for *G. aurea* [63,64]. A close relative with a minute genome, *Utricularia gibba*, was also found to have reduced intron length and fewer introns per gene [74].

While *Genlisea* is a model system for studying genome size evolution [71], the current published draft genomes are highly fractionated and incomplete, limiting their use in genome structure analysis (as with many of the other carnivorous draft genomes) [75]. *Utricularia*, the sister clade to *Genlisea* within the Lentibulariaceae, also contains minute genomes. While the fold-difference in genome size is much less than in *Genlisea*, *U. gibba* is a much more complete and contiguous genome for comparative analyses [62]. *U. gibba* has undergone at least two WGDs in its evolutionary history beyond the whole genome triplication (WGT) event at the base of all core Eudicots [62,74]. Despite having its genome duplicated at least two additional times during its evolutionary history, *U. gibba* has one of the smallest known plant genomes, with its assembled haploid genome size being about 101 Mbp (estimated haploid genome size 77–103 Mbp [72,73,74]). Conversely, *Cephalotus follicularis*, another carnivorous taxon, has not undergone any additional WGDs since the core Eudicot WGT [70] and has an estimated haploid genome size of at least 1.98 billion base pairs (Gbp) [76] (although the haploid genome size of this species was previously reported at 625 Mbp [77]). Like the *Genlisea* example, the size differences between these genomes are primarily due to silencing and sloughing off [74] or proliferation of intergenic (i.e., TE) content [70]. It was proposed that genomes with strong DNA deletion bias, like that of *G. nigracaulis* and *U. gibba*, through WGDs duplicating the gene content, may protect against the loss of essential genome sequences [64,74]. Additionally, it was proposed that genome size evolution may be selectively neutral because repeat content (i.e., TEs) appears to be dispensable in smaller genomes, therefore not serving a functional role in the genome [64]. While *Utricularia* and *Genlisea* can be model systems for studying genome size evolution, carnivory alone does not significantly affect the decreases we see in genome size [76]. It has been hypothesized that a unique cytochrome c oxidase (COX) mutation in *Utricularia* and *Genlisea* may be important for the reduction in genome sizes through increasing the generation of reactive oxygen species (ROS), which damage DNA through point mutations and double stranded breaks [78]. When this was investigated, there was no significant correlation between the COX mutation and genome shrinkage [76].

WGDs have been hypothesized to be a mechanism for plant survival and evolution under stressful biotic and abiotic conditions by providing gene redundancy for evolutionary forces to act on [79]. Another mechanism for introducing gene redundancy is through small-scale gene duplication. This kind of gene duplication has been analyzed for *U. gibba* [62], *U. reniformis* [65], and *C. follicularis* [70]. For *U. gibba*, defense-related, nutrition acquisition, and stress response genes contained large expansions. Among these were tandemly duplicated genes with trap-specific or trap-enhanced expression [62]. These include genes possibly involved in active bladder movements, the breakdown of prey (i.e., cysteine proteases), and the transportation of nutrients. In contrast to the larger *U. reniformis*, the two genomes displayed distinct deletion, duplicated gene, and rearrangement patterns. While sharing some functional enrichments, they embarked on divergent evolutionary paths following the species split, including *U. reniformis* undergoing an additional WGD [65]. These differences, in part, may be the result of selection pressure for adaptation to aquatic habitats for *U. gibba* and terrestrial and epiphytic habitats for *U. reniformis*. The genome of *C. follicularis* also contained tandem duplications of genes with trap-dominant expression. These include wax and cutin biosynthesis, wax ester synthase, and aspartic protease genes [70], all of which are important for its carnivorous syndrome.

Whole genome duplication (WGD) and small-scale duplication have also been studied in the genome evolution of *Nepenthes gracilis* [61]. *N. gracilis* has had at least two lineage-specific polyploidy events since the core-eudicot WGT, resulting in its present-day decaploid (2n) structure for the genus. For the haploid genome assembly, *N. gracilis* has five sets of eight chromosomes: a single set of eight dominant subgenomes and four sets of eight recessive subgenomes. While dominant subgenomes had higher levels of gene expression, 47% of syntenic gene pairs had higher expression on recessive subgenomes, including within the male-specific region of the Y-chromosome and small-scale duplicates of *Nepenthes*-specific tissue-specific (i.e., pitcher) genes [61]. The relaxing of purifying selection of gene copies (i.e., *LFY*) and regulatory regions on the recessive subgenomes may have facilitated the evolution of dioecy in *Nepenthes*, the only carnivorous genus with separate male and female individuals, as well as its present-day carnivorous syndrome. A notable example of tissue-specific expression in *N. gracilis* is a massive tandem cluster of *SRG1* genes on a recessive subgenome, which may be involved in scavenging ROS during prey digestion and nutrient absorption [61].

The Droseraceae, which has also been researched in some detail, is one of the largest carnivorous plant families with three morphologically diverse genera: *Aldrovanda*, *Dionaea*, and *Drosera*. All three genera share a family-specific WGD, and *Aldrovanda* has undergone an additional WGT [68]. Despite the additional WGT, *Aldrovanda vesiculosa*’s genome (508–606 Mbp) is about one-sixth the size of *Dionaea muscipula* (2699–3232 Mbp) [68,80]. As with the above examples, TEs were much more abundant in the larger genomes. It estimated that 38.78% of *D. muscipula*’s genome is made up of long terminal repeat (LTR) retrotransposons, a type of TE, while *A. vesiculosa* and *Drosera spatulata* only have contents of 17.5% and 5.7%, respectively. Previously, the LTR content of genome was found to be highly correlated with genome size [81]. Additionally, intron lengths of *D. muscipula*’s were 1.5-fold larger than the other two species. Tandem gene duplication was much more extensive in *D. spatulata*, with genes containing leucin-rich-repeat (LRR) and IQ domains with putative roles in prey perception [68]. Finally, in this family, it appears that ancestral root genes were co-opted for new carnivory-specific roles, and all three species have duplications in genes related to prey attraction, perception, digestion, and nutrient absorption [68].

Carnivorous plants are also marked by gene losses that reveal insights into their evolution. *Utricularia gibba* has been found to be missing peroxidase genes important for reactive oxygen species (ROS) detoxification, which may lead to the damage of biomolecules including double-stranded breaks in DNA [75]. When the molecular machinery repairs double stranded breaks, a bias for DNA deletion in the repair process may lead to genome shrinking. Furthermore, *U. gibba* was found to be missing several DNA repair genes [75]. Substitution rates in nuclear, plastid and mitochondrial DNA sequences have been shown to be higher in *Utricularia*, *Genlisea*, and *Pinguicula* compared with non-carnivorous relatives [19,82,83,84]. The combination of ROS damage and decreased DNA repair capabilities may lead to the increased mutation rates observed in *Utricularia*. Furthermore, WGDs may be more prominent in *U. gibba* due to a putatively missing member of the JASON gene family [75]. Mutants for this gene in Arabidopsis thaliana result in triploid progeny, so it is possible that *U. gibba* has a genomic bias toward polyploidy events. Finally, a particularly remarkable aspect of *U. gibba* is its absence of roots. Numerous genes associated with root and shoot growth and development are notably absent from the genome of *U. gibba* [75].

The Droseraceae has also experienced gene loss. *A. vesiculosa*, *D. spatulata*, and *D. muscipula* are three of the most gene-poor genomes to date [68]. It has been suggested that because members of this family started to derive nutrients from animal prey, purifying selection for genes important for non-carnivorous nutrition was reduced, leading to massive gene losses. Some of these losses were in genes involved in kinetochore formation and shown to be related to the presence of holocentric chromosomes. Additional losses were in stress responses and root development. Notably, *A. vesiculosa* lacked any key regulators of root development, which is likely related to the adult plant lacking a root system altogether [68].

Finally, Carnivorous plants are excellent examples of convergent evolution. For example, digestive fluid proteins were compared between *Cephalotus follicularis*, *Drosera adelae*, *Nepenthes alata*, and the purple pitcher plant, *Sarracenia purpurea*, in the Sarraceniaceae (Ericales) [70]. The digestive fluid proteins for these four species grouped more closely than expected on the protein trees, despite having three independent origins for carnivory (*Drosera* and *Nepenthes* share a carnivorous origin). This suggests that the same ancestral orthologous genes between these species have been repetitively co-opted for the evolution of carnivory in different plant lineages through convergent evolution [70]. Botanical carnivory offers a clear case of convergent evolution seen in RNase T2 enzymes. Even though *Utricularia gibba* and *C. follicularis* are distantly related, they both show shared functional enrichments for RNase T2 enzymes, which are well-known elements of trap secretions [70]. Additionally, *C. follicularis* and *N. alata* display significant convergent amino acid changes in RNase T2s, purple acid phosphatases, and GH19 chitinases. Similarly, RNase T2s also show substantial convergent amino acid changes in *C. follicularis*, *N. alata*, *Drosera adelae*, and *D. muscipula* [70].

## 9. Conclusions

The relationships shown in the Angiosperm-353 phylogeny mostly support previous plastid DNA studies that show the position of carnivorous taxa form divergent clades sister to large and diverse groupings of multiple families ([40], reviewed in [9]). In combination with the previous plastid studies, this nuclear DNA perspective allows for more informed genomic and proteomic investigations of evolution and development within and between carnivorous plant lineages. Understanding the family and generic relationships also provides a firm foundation for studies of biogeographic history, molecular dating, and morphological evolutionary patterns across angiosperms. Future studies that expand the sampling of whole chloroplast genomes may provide additional support for relationships outlined in the current review. The incongruence observed between the available full chloroplast and the current nuclear Angiosperm-353 genomic topologies requires further investigation, particularly regarding the phylogenetic position of Lentibulariaceae and the monophyly of *Drosera*. Lastly, despite only having a few genome assemblies available for study, carnivorous plant genomes provide important insights into genome evolution. Limitations on this topic are a consequence of the highly fragmented and incomplete state of these assemblies. For future research, more complete and chromosome-level genomes, such as the chromosome-level *Nepenthes gracilis* genome assembly, are needed for high-confidence comparative analyses between various carnivorous and non-carnivorous plants.

## Data Availability

No new data were created or analyzed in this study. Data sharing is not applicable to this article.
